# Correction: Vitamin D levels and risk of type 1 diabetes: A Mendelian randomization study

**DOI:** 10.1371/journal.pmed.1003624

**Published:** 2021-04-29

**Authors:** Despoina Manousaki, Adil Harroud, Ruth E. Mitchell, Stephanie Ross, Vince Forgetta, Nicholas J. Timpson, George Davey Smith, Constantin Polychronakos, J Brent Richards

There is an error in reference 24. The correct reference is: Diamond Project Group. Incidence and trends of childhood Type 1 diabetes worldwide 1990–1999. Diabet Med. 2006;23(8):857–66. Epub 2006/08/17. pmid:16911623.
