# Supplementary material for: Vitamin D levels and risk of type 1 diabetes: A Mendelian randomization study
Source: PLoS Med. 2021 Feb 25;18(2):e1003536. doi: 10.1371/journal.pmed.1003536 (PMC7906317; doi:10.1371/journal.pmed.1003536)
Supplement: Attachment — Submitted filename: Rebuttal_Plos_Med_AH_REM_njt.docx [file pmed.1003536.s006.docx]

Dear Dr Turner,

Thank you and the reviewers for your comments and constructive critique and for the opportunity to resubmit our manuscript. We have addressed the editors’ and reviewer’s comments and the formatting issues, and we hope that the present improved version is acceptable for publication in *PLOS Medicine.* Our answers to the comments of the editors and the reviewers appear in blue.

Requests from the editors:

We note that one reviewer mentions STROBE-MR. As far as we are aware, this has not yet been published in peer-reviewed form, and so we are not requesting that authors of relevant studies use that guideline. 

We suggest adding "levels" to the title.

We have added "levels" to the title as suggested, which is now "Decreased vitamin D **levels** and risk of type 1 diabetes: A Mendelian randomization study".

Please add a new final sentence to the "Methods and findings" subsection of your abstract, quoting 2-3 of the study's main limitations.

We added the following phrase at the end of the Methods and findings paragraph of the abstract: “Our study was sufficiently powered to exclude large effects (OR> 1.23), thus small effects cannot be excluded.”

Please add "Our findings suggest that ..." or similar to begin the "Conclusions" subsection of your abstract.

We modified the phrasing of the Conclusion section of the abstract according to the editor’s suggestion.

After the abstract, please add a new and accessible "author summary" section in non-identical prose. You may find it helpful to consult one or two recent research papers published in PLOS Medicine to get a sense of the preferred style.

We added the author summary as requested by the editor.

Throughout the paper, please adapt reference call-outs to the following style: "... [8]." (i.e., consistently preceded by a space and preceding punctuation, and without spaces within the square brackets).

We adapted the citation style according to the editor’s suggestions.

Early in the methods section of your main text, please state whether the study had a prespecified analysis plan or protocol, and if so attach the document(s) as a supplementary file(s), referred to in the text. Please highlight analyses that were not prespecified.

There was no prespecified protocol or analysis plan for this study. A relative statement appears at the beginning of the Methods section. We highlighted the parts of the methods section as requested.

Please note in the Methods section that ethics approval was not required for this study (or quote the authority and ethics approval).

We added an ethics statement at the beginning of the Methods section.

At the start of the Discussion section, please rephrase "... failed to support ...".

We rephrased the start of the discussion (page 16, line 369), as follows: “The results of this MR study do not support a causal association..”

Throughout the text, please quote p values alongside 95% CI, where available.

We added the p-values after the 95% CIs as requested. 

Please remove the information on funding from the end of the main text. In the event of publication, this information will appear in the article metadata, via entries in the submission form. 
The funding information was removed.

Noting reference 1 and some others, e.g., reference 26 and 27, please ensure that all references quote full access details.

We corrected the references and added DOI, PMID and PMCID to each reference.

Please spell out the institutional author name for reference 22, and add access details.

We added the full reference: Canadian Diabetes Association. An economic tsunami: the cost of diabetes in Canada. December2009.https://www.healthyenvironmentforkids.ca/sites/healthyenvironmentforkids.ca/files/Economic_Tsunami.pdf

Please add a completed checklist for the most appropriate reporting guideline, which we suspect will be STROBE, as a supplementary document, referred to in the methods section (e.g., "See S1_STROBE_Checklist"). In the checklist, please refer to individual items by section (e.g., "Methods") and paragraph number rather than by line or page numbers, as the latter generally change in the event of publication. 
We added the STROBE checklist as a Supplementary document.

Comments from the reviewers:

*** Reviewer #1: 

The data is sensibly chosen and the analysis method is well done and sufficient to warrant their conclusion. The method is explained in sufficient detail in the main text - some of this could be summarized to make a more streamlined paper, and the detail moved to a supplementary section - and should be reproducible by anyone with access to the data. 
The paper is sensibly laid out and should be possible for a interested non-specialist to follow. 

No mention is made of the STROBE-MR guidelines, but the paper has followed all the major points. 
The STROBE-MR checklist has not been peer-reviewed, and thus does not appear in the list of official checklists for manuscripts. Instead, we included the STROBE checklist.

Figure 2 shows a convincing lack of any pattern, supporting the null result found; however it would be clearer to restrict to just the major method used in the paper (ivw, median, mode, mr-egger) and to add error bars to the points.

We modified Figure 2 to only include the Methods outlined in the paper.

I think the power calculations are slightly underestimated. The numbers given in the paper give me 80% power to detect an effect of size 1.23, and 87% power to detect 1.25 (from <https://cnsgenomics.com/shiny/mRnd/>)

The reviewer is right and we thank him/her for picking this up. We modified the OR in the results and discussion section accordingly: page 15, line 364: “our study had a power of 80% power to exclude effects on type 1 diabetes as small as an OR of 1.23 per one standard deviation change in 25OHD on the log scale” and page 16, line 372: “Our findings can confidently exclude large effects (OR >1.23), given the sufficient statistical power and the consistency of the estimates using different MR methods and in the sensitivity analyses”

The discussion section needs to include a wider discussion of the current literature. This null result has been shown before in <https://academic.oup.com/ije/article/48/5/1425/5569493>"Phenome-wide Mendelian-randomization study of genetically determined vitamin D on multiple health outcomes using the UK Biobank study" (This is easy to miss as the null results are discussed individually in the main body of the paper, but Type 1 diabetes is one of the outcomes in MR-PheWAS and it was no significant as show in this image <https://academic.oup.com/view-large/figure/178161527/dyz182f2.tif> ) . The instrument they used was weaker (2.84% of trait variance explained using 6 snps) and this confirmation of that result is useful. Clarification as to the degree of overlap in individuals in the outcome datasets & SNPs under consideration between the two studies would be helpful.

We thank the reviewer for bringing this to our attention. In the Meng et al PheWAS paper, a polygenic risk score composed by the 6 25OHD SNPs was initially tested for association with 920 outcomes with more than 200 cases in UK Biobank (we suspect that type 1 diabetes was among these 920 outcomes). The 9 outcomes for which a positive association was detected where further investigated in formal MR studies- and type 1 diabetes was not one of these 9. We could not find information in the manuscript by Meng et al on the exact number of type 1 diabetes cases and controls in UKBB. The diseases/traits with null outcomes among the 920 tested are not outlined in the main paper or the supplement. Thus we elected not to include the Meng et al study in our discussion, since it is not clear in the paper that type 1 diabetes was among the outcomes tested for association with 25OHD levels.

A greater prominence should also be given to a discussion of how differing geographic areas of studies may impact the SNPS results. "Variation in Associations between Allelic Variants of the Vitamin D Receptor Gene and Onset of Type 1 Diabetes Mellitus by Ambient Winter Ultraviolet Radiation Levels: A Meta-Regression Analysis" (<https://academic.oup.com/aje/article/168/4/358/106033> ) showed that impact of SNPs on T1D varies with UV levels, suggesting that the impact in vitamin D deficient groups differs from that in non-deficient population. How homogeneous is the population under consideration in terms of geographic location? This could also be indicative of a non-linear effect. The authors say that traditional MR methods assume linearity, but there are a range of techniques adapting MR to non-linear situations - <https://pubmed.ncbi.nlm.nih.gov/28317167/> or <https://www.bmj.com/content/364/bmj.l1042> . If the authors have appropriate data to apply these, they should be
considered. 
We thank the reviewer for this suggestion- we added the reference and a phrase in our discussion section (page 19, line 431): “Despite the fact that the SNPs used as instruments in our MR were extracted from GWAS in Europeans, the populations of both GWAS were not homogenous in terms of geographic location. It has been shown that there could be some gene-environment interaction in the effect of SNPs in the vitamin D receptor gene on type 1 diabetes risk [49]. Specifically, the effect of these SNPs on type 1 diabetes varies with levels of ultraviolet irradiation, suggesting that the impact in vitamin D deficient groups differs from that in non-deficient population. This raises a possibility of gene-environment interaction for SNPs affecting 25OHD levels and of non-linear effects of these SNPs on risk of type 1 diabetes, but two-sample MR studies can only assess linear associations.”

However, in order to perform the suggested method to test for non-linear effects, estimation of the exposure‐outcome relationship requires both individual‐level data and a one‐sample setting in the MR (otherwise neither stratification of the population nor the estimation of genetic associations with the outcome in the strata is possible). Thus it is impossible to undertake this analysis in a two-sample MR setting.

Under limitations, the authors should also explicitly discuss the lack of applicability to other ethnic groups, and potentially other geographic areas. 
We added the following phrase in our limitations section in the discussion (page 19, line 445): “Our MR results cannot be generalized to non-Europeans and potentially to Europeans residing in different geographic areas than those of the participants in the vitamin D and type 1 diabetes GWAS.”

*** Reviewer #2: 


COMMENTS

1.Presentation of existing evidence from observational evidence: There is a relatively large literature linking various aspects of vitamin D with T1D, but my assessment is that the authors have not done a balanced assessment / selection of papers to discuss. I would suggest that the authors focus on available prospective studies of 25OHD in the current context. "Cross-sectional" studies comparing 25OHD in people diagnosed with T1D compared to controls are not high-quality epidemiological evidence, as these are clearly much more prone to both selection and reverse causation bias (e.g. refs 5-7), than are prospective studies (e.g. ref 9 and 12). One of the largest prospective studies in the field, with longitudinal 25OHD measurements from early childhood is the TEDDY study (Norris, Diabetes 2018). I would say this is a glaring omission from the intro and discussion. While TEDDY/Norris and TRIGR (Miettinen, ref 12) found evidence of an inverse association, the associations were
moderate or weak, and not consistent with Simpson (ref 9) and Raab (ref 11). While islet autoimmunity (IA) is a surrogate endpoint for T1D, it is important to note that prospective studies of 25OHD before IA are less susceptible to reverse causation bias than other study types in the field (but note that Raab measured 25OHD after seroconversion, and is prone to potential reverse causation bias!). Yet another important, prospective study is Makinen/DIPP, J Clin Endocrinol Metab 2016, who found no significant association between longitudinal 25OHD and progression from IA to T1D. Again in the discussion (2nd paragraph), 3 low quality studies are cited to support  observational evidence for a causal role of low 25OHD. I understand that it is nice if the story fits into the "MR narrative" where observational studies show an association and MR not, but a balanced review of existing high quality prospective studies are in my mind not consistently showing an inverse association with
T1D. Finally, in one of the last paragraphs of the discussion, where the authors discuss limitations, ref 3 (Hypponen Lancet 2001) is cited to support that deviation from linear association between 25OHD and T1D should not be a problem. This is either a typo or an incorrect assessment of this paper. Hypponen 2001 did NOT include measures of 25OHD (and all of the association is based on less than 1% of the study population who deviated from the general advice to take vitamin D supplements), and clearly cannot be used to assess linearity of the potential 25OHD-T1D association.

We thank the reviewer for the constructive suggestions and for providing detailed references. We made sure to incorporate the new references in the manuscript, and to underplay the non-prospective studies in our introduction and discussion sections. The literature review parts in the introduction and discussion were rewritten.

In regards to the reference by Hypponen et al, the reviewer is right. This study has tested effects of supplementation with vitamin D at different doses on risk of type 1 diabetes. Thus, we rephrased the part of the discussion as follows (page 19, line 443): “prospective studies have reported a linear relationship between doses of vitamin D supplementation and risk of type 1 diabetes”

When the authors discuss the fact that their study cannot exclude the possibility that prenatal vitamin D status could influence T1D risk, I would suggest to cite two large scale prospective studies of maternal or neonatal 25OHD in relation to T1D (showing relatively precise estimates near null): Thorsen AJE 2018 and Jacobsen, Diabetologia 2016 (I am admittedly a co-author on one of them, so I may be biased, but you should at least assess these papers).

We added the two references in the discussion section (page 18, line 429): “ In this regard, our study could not exclude effects of intrauterine exposure to lower 25OHD levels in the risk of type 1 diabetes in the offspring which have been examined in observational studies”.

Also reference to existing animal studies seems a bit unbalanced in my mind. Ref 8 cited in the introduction is not a study of mice as the authors state, but in vitro studies of human islets. There are many studies of various aspects of vitamin D and diabetes in NOD and other rodent models (in addition to ref 2), of various relevance to humans.

We thank the reviewer for identifying this error. We modified the phrase in the introduction as follows (page 4, line 109): “Additionally, it has been shown that vitamin D could prevent cytokine-induced apoptosis of human pancreatic islets [14]”. We also added references 3,4 and 5 on animal studies.

2. SNPs and effects: I am not a GWAS or MR expert, but impression is that the analyses are competently performed. I have couple of comments which may reflect my of expertise. Given that most of the 69 SNPs were recently identified in UKBiobank, can we be confident that the R-square is not upwardly biased due to overfitting?  I may be wrong, but after browsing the AJHG paper I could not see that the novel SNPs were replicated? I am worried that if the Rsq is overestimated, then so is the statistical power in the current analysis (and effect estimates probably also biased?). If the UK Biobank were the data set used to identify these novel SNPs, the effect estimates may have been biased (cf winners curse), and Rsq should perhaps have been estimated based on a replication dataset or at least cross validation (I may have browsed too quickly over this in the paper or cited reference, but these aspects should probably be better described).

In regards to the question of the reviewer about overfitting: If we understand well, the point raised by the reviewer about overfitting could be a concern in the power calculation for our MR study. This relies in part on the variance explained by the SNPs, which was estimated in the same cohort as the GWAS. The Rsq could therefore be overestimated, and the power calculation overestimated. Unfortunately, we do not have access to an independent cohort where the Rsq could be estimated for those SNPs. We added a phrase in the limitations section of the discussion to underline this (page 19, line 451): “Finally, there is a possibility that our MR power calculation was overestimated due to overfitting caused by the fact that the variance explained by the vitamin D SNPs was calculated in the same population as the vitamin D GWAS. Unfortunately, we do not have access to an independent cohort where the amount of variance explained by those SNPs could be estimated”.

Lack of replication of loci in the UKBB vitamin D GWAS: The sample size of the vitamin D GWAS (~443K), ensured enough statistical power to achieve p-values for most identified loci that were well below the threshold of 1.2 x 10^-9^. In such settings, the discovery GWAS does not need to be necessarily replicated. A separate group has published a vitamin D GWAS in the same UKBB population (Revez et al, Nature Comm 2020) using slight different methods to define the phenotype, run the GWAS and define independent loci. They identified the same amount of independent SNPs and the same loci as the AJHG GWAS. Although we could not argue that the Revez et al GWAS results replicate the results of the AJHG GWAS (since the two GWAS were not in separate populations), these comparable results provide clear evidence of the validity of the identified GWAS loci.

  
3. Population stratification? Again this question may reflect my lack of expertise in MR data analysis, but I wonder whether population stratification is properly adjusted for in the analyses. Even if the populations are of European descent, could not population stratification still confound results, if allele frequency and T1D risk differed systematically across strata? (or was this handled by adjusting each of the SNP-25OHD - and SNP - T1D associations for principal components?).

The reviewer is right. Bias from population stratification in the GWAS are addressed by ensuring that only individuals of the same ancestry (here Europeans) are included in the exposure and outcome GWASes, and by the fact that adjustments for population stratification are performed in the GWAS models (genomic control, principal component analyses). But residual population stratification can still be an issue in a two-sample MR. We included a phrase in the discussion to underline this (page 19, line 447):“although we assured that both exposure and outcome GWAS were restricted to participants of European ancestry, residual confounding from population stratification cannot be completely excluded in a two-sample MR setting, due to ethnic differences in the exposure and outcome GWAS populations [50].”

4. calculation and interpretation of statistical power
The paper cited for power calculations (Brien et al. 2013) is for continuous outcomes, not binary such as T1D. Would't it make more sense to use Burgess' method (IJE 2014), for binary outcomes? It may not make a major difference in practice, but would be good to clarify.

While the reviewer rightly points that the original publication by Brion et al. 2013 applied to continuous outcomes, the authors have since adapted their equations for estimating power using the non-centrality parameter for binary outcomes. This is detailed in the documentation of their online calculator available at <https://shiny.cnsgenomics.com/mRnd/>. At the request of the authors, we have cited the article by Brion et al. 2013 (citation tab of the aforementioned website).

Most importantly, based on the given power of 80% under an alternative hypothesis where the true OR is 1.25, the authors state in the end of the results section and again in the first paragraph of the discussion that "…results are robust to exclude large effects (OR >1.25), given ….". I do not think this is a proper interpretation. The test (or analysis) failed to detect a significant association, but did not exclude the possibility that the true OR is 1.25! 
We would like to bring to the reviewer’s attention that following the comment of reviewer 1, we recalculated the minimal OR to achieve a power of 80%, and it came down to 1.23. We’ve rephrased the parts of the results section as follows (page 16, line 372): “Our findings can confidently exclude large effects (OR >1.23), given the sufficient statistical power and the consistency of the estimates using different MR methods and in the sensitivity analyses.”

MINOR COMMENTS
-a strength in the current study is that all 25OHDs were measured using a single assay (presumably in the same lab). However, the 12 cohorts used for T1D associations could perhaps be described in some more detail (age at onset, selection of controls, control of ethnicity and other types of population stratification, etc. (or did I miss this)

We added the following phrase in the methods section (page 9, line 212): “Details on the demographics of the cohorts participating in the 25OHD and type 1 diabetes GWASes can be found in the respective publications [31, 32].” The information the reviewer is requesting can be found in Table S1 of reference 32. The age at onset of T1D was not available in the T1D GWAS paper.

-The authors explained in the methods section that they presented the results in terms of a given contrast in 25OHD, which is important. It would probably help the readers if this was presented right away in the methods where this is explained (and perhaps briefly mentioned in the abstract too; it is after all not a major result).

Although providing the exact amount of change in nmol/l of 25OHD levels corresponding to a 1SD decrease is clinically relevant, this amount of change is different among vitamin D deficient, insufficient and sufficient individuals. We elected thus to present in the abstract only what this change represents in vitamin D insufficient individuals:

“MR analyses demonstrated that a one standard deviation decrease in standardized natural log-transformed 25OHD (corresponding to a 29 nmol/l change in 25OHD levels in vitamin D insufficient individuals) did not increase type 1 diabetes risk (inverse-variance weighted MR OR=1.09, 95% CI: 0.86-1.40, p=0.48).”

- While the authors have done a great job at assessing potential pleiotropy, and also discussing potential limitations of the study, my impression is that even with the "traditional" vitamin D pathway SNPs, we do not really know much about the functional consequences of the polymorphism (the fact that they are near a relevant enzymes is of course reassuring, but not enough).  Furthermore, one of the loci most robustly associated with 25OHD is GC (encoding Vitamin D-binding protein, DBP), which in addition is also strongly associated with circulating DBP-concentrations. It is admittedly not well established, but a role of DBP in the aetiology of T1D has been suggested in a few studies.

We agree with the reviewer that residual pleiotropy cannot be excluded in the absence of information on the function of each of the SNPs used as instrumental variables in a MR study. Since the number of 25OHD SNPs is now large enough to allow application of different statistical methods sensitive to pleiotropy, we elected to apply a variety of these methods, in order to identify pleiotropic pathways. The results of all methods showing null results were consistent. To specifically respond to the reviewer’s comment on the GC SNP, the leave-out method (MR-PRESSO), did not identify the GC SNP as being an outlier (see Supplemental Table 3).

-The first reference cited for incidence of T1D in Canada, is incorrect (reference is about something else), probably a typo. Personally, I would cite a reference showing the incidence not only in a single country, and some of the variation across the world and over time.

We rephrased the beginning of the introduction, adding a new reference (page 4, line 101): Type 1 Diabetes is a relatively common autoimmune disease… its incidence is increasing worldwide [1]

-Ref 19 is cited for expected risk in prevalence of T1D worldwide, but ref 19 is about something else! (genetic interactions)
We thank the reviewer for spotting this error. We replaced the erroneous reference (now reference 24) by a correct one.

-in the methods section, the authors state that they calculate risk associated with a SD increase, while the actual results were in fact presented as OR for an SD decrease.

We rephrased the section in the methods as follows (page 8, line 200): “To assess whether genetically lowered vitamin D levels are associated with increased odds of type 1 diabetes”.

-Ziegler ref 18 on skin pigmentation. Could this observation not simply have been due to ethnicity? I am not sure this is convincing evidence for reverse causation.

We agree with the reviewer that we cannot be certain about the link between less skin pigmentation and sun exposure. We rephrased the related phrase as follows (page 6, line 150): “Also, subjects affected by type 1 diabetes are more sensitive to ultra-violet radiation and have less skin pigmentation than healthy controls [23], which could suggest that reduced sun exposure might account for the low level of vitamin D in these patients.”

*** Reviewer #3: 

1.	This might seem pedantic, but can the authors provide one more sensitivity analysis ? Using the 4 "canonical" SNPs rather than the 6 in the Jiang et al study. I ask for two reasons - the 6 SNPs in Jiang et included 2 outside of the key 4 that lie near key Vit D metabolism and synthesis genes, and it makes sense to exclude these from this very specific test. Second, the Cooper et al study in 2011, did see a positive association with these canonical variants, in what appears to be a very similar set of T1D cases. Third, fig 2 shows nicely how a very small number of much more specific-to-vitamin-D SNPs could have a big influence on the MR result.

As requested by the reviewer, we undertook a sensitivity analysis using only the 4 previously known 25OHD SNPs, in genes directly involved in vitamin D synthesis and metabolism. The IVW results did not differ from those of the main analysis with 69 SNPs or those of the analysis with 6 SNPs. We now have added this analysis in the Methods and Results sections:

Page 12, line 283: “Lastly, to further ensure that our estimates were not influenced by pleiotropy, we repeated the MR analysis using only 6 genetic variants for 25OHD levels identified in a previous GWAS, and also undertook a separate analysis using 4 among these 6 SNPs, which lie in or next to the *DHCR7, CYP2R1, GC, CYP24A1* genes which directly regulate vitamin D synthesis or degradation [39]”

Page 15, line 360: “When we limited our MR instruments to only 4 among these 6 SNPs (which involve genes with direct role in vitamin D synthesis and metabolism), the results were similar (IVW MR OR=0.92, 95% CI:0.53-1.62, p=0.78).”

2.	Many tests show considerable heterogeneity and some MR egger intercept p values suggest there could be meaningful levels of pleiotropy that are hard to properly account for. Many of these pleiotropic effects likely reflect the fact that most SNPs have strong effects on traits other than vitamin D. Although the methods used adjust the models for such effects, none can eliminate all the sources of error. Ultimately, the most useful advance for this question would be a larger sample size for the outcome trait - T1D cases and controls. Given that many of the MR results are trending towards a positive effect of Vit D in protecting from T1D, the question is crying out for a larger sample size.

We agree with the reviewer that larger T1D GWAS would provide better power in future MR studies, to exclude even small effects-something that cannot be definitely excluded by our MR study.

Interestingly, reviewer 1 brought to our attention that in the study by Meng et al (IJE 2019), the association of a polygenic risk score for vitamin D levels with T1D was tested in UKBB, and the results were null. Although we suspect that the number of cases of T1D in UKBB are less than in the Forgetta et al GWAS, this evidence provides support to our MR findings.

Minor points:
1.	Can a study be biased by confounding ? I think confounding is confounding ? likewise reverse causation is reverse causation , not bias ?

We rephrased the introduction and discussion section to remove the term “bias” when discussing confounding and reserves causation as limitations of observational studies.

2.	Suggest avoid chatty phrases such as "Widely known" and "on the other hand"

We agree with the reviewer- we replaced the chatty phrases using more formal terms.

3.	The background oversells MR a little - it is not immune from many of the issues you describe, such as reverse causation, confounding and not entirely equivalent to an RCT. Suggest qualify.

Following the reviewer’s suggestion, we downplayed the MR strengths in the introduction and discussion sections of the manuscript.

Page 7, line 177: “Mendelian randomization (MR) is a method in genetic epidemiology which uses genetic variants reliably associated with exposures of interest to estimate causal associations between a given biomarker, such as vitamin D, and disease. These studies have orthogonal assumptions to conventional techniques and are arguably less prone to reverse causation since disease states usually do not change the germline DNA sequences [29]. Most importantly, MR can limit confounding, since genotypes are randomly assorted at meiosis [30]. In this regard, the MR framework can be compared to that of an RCT because the random assortment of genetic variants replicates the random allocation of study participants to different therapeutic arms.”

Page 17, line 396: “Our MR approach has several strengths. First, its design decreases potential confounding or reverse causation which are present in observational studies.”
