# Supplementary material for: Vitamin D levels and risk of type 1 diabetes: A Mendelian randomization study
Source: PLoS Med. 2021 Feb 25;18(2):e1003536. doi: 10.1371/journal.pmed.1003536 (PMC7906317; doi:10.1371/journal.pmed.1003536)
Supplement: Attachment — Submitted filename: Rebuttal.R2[1].BR.docx [file pmed.1003536.s007.docx]

Dear Editor,

We thank you and the reviewers for your feedback, and for the opportunity to resubmit our manuscript. Below you will find our responses to your comments in blue. We hope that the present version of our manuscript will satisfy the criteria for publication in your journal.

Sincerely,

Despoina Manousaki

*The editors felt it would be good to also mention (briefly) additional possible limitations in the abstract (as are noted in the full discussion section, eg the possibility for pleiotropy and canalization, as well as the point already noted that very small effects can't be excluded.

We thank the Editor for this suggestion. We added the following phrase in the abstract:

“Our study was able to reasonably exclude large effects outside the 95% confidence intervals (OR > 1.40) thus we could not exclude small effects. We also cannot provide insights into the effect of 25OHD in people with extremely low 25OHD levels and in persons of non-European ancestry”

*Many thanks for including the information about the lack of a prespecified analysis plan per journal policy - however, the point that "group has a long track of published MR studies" could probably be excluded.
We removed this sentence as requested.

*The funding statement in the main manuscript text can be deleted, as this is provided in the submission form (and needs only to be there, not in the actual manuscript as well). 
The funding statement was removed from the main manuscript.
-----------------------------------------------------------

Comments from the reviewers:

Reviewer #1: I disagree with the author's choice not to discuss Meng et. al. paper because the "diseases/traits with null outcomes among the 920 tested are not outlined in the main paper or the supplement". It is clear from the paper (and supplement) that they have used the mapping from ( <https://phewascatalog.org/phecodes> ) which includes Type 1 diabetes, this can also be seen in reference 8 of the main paper ( <https://academic.oup.com/bioinformatics/article/30/16/2375/2748157> ). Given the concern over exact case numbers, they could contact the authors. However the UK Biobank website shows it would be ~ 1000 people ( <https://biobank.ctsu.ox.ac.uk/crystal/field.cgi?id=41202> ). 
This paper is a clear improvement on that study, as it is focused exclusively on type 1 diabetes and thus able to discuss and investigate the null result in detail, but I still feel adding the confirmation of this result in other sources is important - particularly when they use it to respond to reviewer 3's comments! 

Otherwise I think this paper has responded well to my comments and those of the other reviewers, and is ready to publish.

We agree with the reviewer that all previous evidence in this field should be presented in our manuscript. Therefore, we cited the Meng et al study in the discussion (line 389, page 17) : “More recently, a phenome-wide association study by Meng et al [45] examined associations between a polygenic risk score for 25OHD levels comprising 6 SNPs and 920 phenotypes in UK Biobank, among which was type 1 diabetes. The authors found no evidence for positive association of 25OHD with type 1 diabetes.”
-----------------------------------------------------------

Reviewer #2: The authors have responded and revised well, with one minor exception. I am not entirely happy with the interpretation of the power calculation. My main point was not about the aboslute power (now 80% power to detect OR of 1.23), but how such a power analysis is interpreted. The authors still say the study "excluded ORs larger than" the calculated smallest OR with 80%. I do not think this is formally correct. If anything, it may be reasonable to say that the study (reasonably) excluded ORs outside the 95% CI. THe fact that you had 80% power to detect true ORs of 1.23 or larger, and you failed, does not mean you excluded such values. At least this is how I have understood this. I am willing to consider a counterargument, but the authors did not really address this in their response. 
The reviewer is correct. The 95% confidence intervals allow us to state that if we re-sampled the population numerous times, the fraction of calculated confidence intervals that encompass the true OR would be 95%. This makes it quite unlikely that the true effect size is clinically relevant (ie that the OR is greater than 1.40, which is the upper CI of our MR estimate).

While we kept the original power calculation in the methods, we rephrased the abstract to reflect this change: “Our study was able to reasonably exclude large effects outside the 95% confidence intervals (OR > 1.40) thus we could not exclude small effects” and we changed the results section (page 15, line 363): “Based on a sample size of 25,063 individuals and setting alpha to 0.05, and the variance explained to 3.1%, our study had a power of 80% power to exclude effects on type 1 diabetes as small as an OR of 1.23 per one standard deviation change in 25OHD on the log scale and a power of 100% to exclude effects as small as an OR of 1.4.”

-----------------------------------------------------------

Reviewer #3: Many thanks for doing that extra 4 SNP analysis.  It is slightly worrying how this result differs from the 2011 result, given the very similar set of outcome cases and SNPs - you might expand a little on why you think the result differs when using the same SNPs and v similar outcome data. I realise they didnt do 2 sample MR so did not accoutn for the SNP-exposure dosage - and I see that the strongest Vitamin D SNP is flat in your data and pulling any protective effect towards the null, but is this one that was associated in the Cooper et al study and what are the differences ?
The reviewer is raising an interesting point. Indeed, when using as instruments the 4 SNPs and their estimates on 25OHD from previous smaller GWAS (for instance the Canadian Multicenter Osteoporosis Study-CaMos, n=2,347) and estimates on T1D from the WTCCC GWAS, we have an MR estimate (considering a fixed-effects model) which is significant, and driven by the *DHCR7* and *CYP2R1* SNPs exclusively. This implies possible violation of the MR assumptions in some of the MR instruments (SNPs). Indeed, the DHCR7 SNP has been associated with geographic region (see PMID 22629401) which is a confounder in the association between 25OHD levels and type 1 diabetes, and when excluded, the positive MR association disappears. Similarly, while the *GC* SNP (which was the SNP with the largest effect on 25OHD levels in the Cooper study), did not have any effect on T1D in this study, the *DHCR7* and *CYP2R1* SNPs drove the reported association.

The advantage of using a larger number of SNPs for 25OHD levels, is that our MR instrument is stronger, since it explains a larger portion of the variance explained in the exposure (25OHD levels). Larger 25OHD GWAS studies offer more precise estimates from known SNPs from previous GWAS. Finally, using MR statistical methods (such as the MR-Egger), the detection of unbalanced pleiotropy among the instruments has become possible in our study.

Considering all the above, we tend to believe that the association between 25OHD levels and T1D described in the Cooper et al paper (which is not an MR study) reflects the presence of confounding in one or more among the used 25OHD SNPs.

We modified the discussion section to comment on this (line 393, page 17):

“The three aforementioned studies do not fulfill the criteria of an MR approach. Also, since these studies were published, our knowledge on vitamin D genetics was substantially expanded with the publication of large vitamin D GWAS meta-analyses [31], providing more precise estimates and a new set of 25OHD SNPs explaining a larger portion of the variance in 25OHD levels.”

-----------------------------------------------------------
