# Supplementary material for: Vitamin D levels and risk of type 1 diabetes: A Mendelian randomization study
Source: PLoS Med. 2021 Feb 25;18(2):e1003536. doi: 10.1371/journal.pmed.1003536 (PMC7906317; doi:10.1371/journal.pmed.1003536)
Supplement: Attachment — Submitted filename: Rebuttal_R3.docx [file pmed.1003536.s008.docx]

Dear Editor,

Thank you for the opportunity to publish our work in Plos Medicine. In the present version, we have addressed the requests from the Editors, and have amended the manuscript to address the comment of the 3^rd^ reviewer. Please find below our answer to the reviewer in blue.

We hope that the revised version will satisfy the criteria for publication in your journal.

Sincerely,

Brent Richards

Reviewer 2:

“thanks for the extra info about the 4 SNP instrument. Sorry to prolong the discussion, but i think the fact that you have estimated the exposure effects in 430k people rather than 2k people could be an explanation for the different results, rather than the stronger instrument - you say that you explain more variance in vitamin D but you have also added in a lot more pleiotropy - and pleiotropy that might not be properly accounted for by MR egger - especially if the INSIDE assumptions are violated - which is possible if there is a consistent dose response effect of lipid SNP > vitamin D levels as well as SNP > lipid levels. Can you say how much more variance you explain compared to the 4 canonical SNPs in your added discussion paragraph , and point out the that Egger is not a perfect solution to pleiotropy ?”

We agree with the reviewer that the MR Egger might have not addressed sufficiently pleiotropy in this study. We added the following phrase in the discussion (line 418): “Even in the case that pleiotropy was not properly accounted for by MR egger (if the INSIDE assumption was violated by some of the SNPs used as instruments), the fact that we obtained consistent results using another two pleiotropy robust MR methods and in sensitivity analyses excluding SNPs with pleiotropic effects is reassuring.” We also added the information on the variance explained of the four SNPs in the results session (line 362): “When we limited our MR instruments to only 4 among these 6 SNPs (which involve genes with direct role in vitamin D synthesis and metabolism **explaining 2.4% of the variance in 25OHD levels**), the results were similar (IVW MR OR=0.92, 95% CI:0.53-1.62, p=0.78).”
